# De Witt County Medical Society

**Published:** 1860-08

**Authors:** Christopher Goodbrake

**Affiliations:** Secretary


On motion, the Society adjourned.

G. W. PHILLIPS, M. D.,

*Secretary.*

### DE WITT COUNTY MEDICAL SOCIETY.

The Society met in quarterly session, at the office of Dr. Richards, at Santa Anna, on the 3d day of July, 1860, at 10 o'clock A. M. Present, Drs. J. H. Tyler, Evan Richards, John McHugh, John Wright, H. S. Norris, Stephen T. Noble and Christopher Goodbrake; also Dr. Fisher, of Le Roy, and Messrs. Edwin Gideon and Rolla T. Richards, medical students, with a goodly number of the citizens of Santa Anna. Dr. John H. Tyler in the chair.

The minutes of the last meeting were read and approved.

On motion of Dr. McHugh, Dr. Fisher, of Le Roy, McLean Co., was invited to participate in the proceedings of the meeting.

Dr. Norris reported a singular case of spasms of an unusual character, in a boy about 15 years of age. From the description as given by Dr. Norris, Dr. Goodbrake supposed masturbation to be the cause.

Dr. Stephen T. Noble read a very interesting essay on "Puerperal Peritonitis," which was discussed by most of the gentlemen present; and on motion, Dr. Noble was requested to furnish a copy for publication.

Dr. Richards read a translation from the French "On Experimental Researches on the Physiology of the Medulla Oblongata," by Brown-Sequard. Also a translation from the French, by Brown-Sequard, "On the Independence of the Vital Properties of the Motor Nerves.

The pathology of fevers was then discussed by most of the members present.

At 4 o'clock the Society adjourned to a grove adjoining the town, to hear an address by Dr. Richards, on "Common Sense, and the Science of Medicine."

After returning to the room Drs. Adams and Edmiston, were continued to read essays at the next meeting.

Cholera Infantum was chosen for the subject of discussion the next meeting.

On motion of Mr. Gideon, the thanks of the Society were voted to Dr. Hugh & Richards, and their ladies, for the very excellent dinner served up for the members of this Society, at the present meeting.

On motion, the Society adjourned to meet in semi-annual session, in the town of Wapella, on the first Tuesday of October next, at 10 o'clock A. M.

CHRISTOPHER GOODBRAKE,

